# 2-{(*E*)-1-[2-(4-Nitro­phen­yl)hydrazin-1-yl­idene]eth­yl}benzene-1,3-diol

**DOI:** 10.1107/S1600536812005399

**Published:** 2012-02-10

**Authors:** R. Alan Howie, James L. Wardell, Solange M. S. V. Wardell, Edward R. T. Tiekink

**Affiliations:** a Department of Chemistry, University of Aberdeen, Meston Walk, Old Aberdeen AB24 3UE, Scotland; b Centro de Desenvolvimento Tecnológico em Saúde (CDTS), Fundação Oswaldo Cruz (FIOCRUZ), Casa Amarela, Campus de Manguinhos, Avenida Brasil 4365, 21040-900 Rio de Janeiro, RJ, Brazil; c CHEMSOL, 1 Harcourt Road, Aberdeen AB15 5NY, Scotland; d Department of Chemistry, University of Malaya, 50603 Kuala Lumpur, Malaysia

## Abstract

The title compound, C_14_H_13_N_3_O_4_, is close to planar, the dihedral angle between the terminal benzene rings being 5.80 (16)°; the nitro group is coplanar with the benzene ring to which it is bonded [O—N—C—C torsion angle = −177.3 (3)°]. The hy­droxy group forms an intra­molecular hydrogen bond with the imine N atom, and the conformation about the imine bond is *E*. In the crystal, layers in the (101) plane with an undulating topology are formed by O—H⋯O and N—H⋯O hydrogen bonds along with C—H⋯O inter­actions. Centrosymmetrically related layers are connected *via* π–π inter­actions [ring centroid–centroid distance = 3.5739 (19) Å] into double layers.

## Related literature

For background on the influence of substituents upon the supra­molecular structures of hydrazones, see: Glidewell *et al.* (2004 ▶); Ferguson *et al.* (2005 ▶); Wardell *et al.* (2007 ▶); Baddeley, de Souza França *et al.* (2009 ▶); Baddeley, Howie *et al.* (2009 ▶); de Souza *et al.* (2010 ▶); Howie, da Silva Lima *et al.* (2010 ▶); Howie, de Souza *et al.* (2010 ▶); Nogueira *et al.* (2011 ▶); Howie *et al.* (2011 ▶).
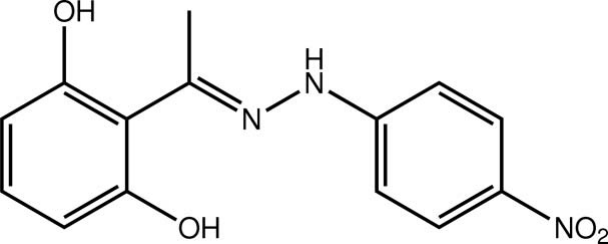


## Experimental

### 

#### Crystal data

C_14_H_13_N_3_O_4_
*M* *_r_* = 287.27
Monoclinic, 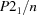

*a* = 7.9714 (3) Å
*b* = 13.5021 (7) Å
*c* = 12.1081 (5) Å
β = 90.186 (3)°
*V* = 1303.20 (10) Å^3^
*Z* = 4
Mo *K*α radiation
μ = 0.11 mm^−1^
*T* = 120 K
0.10 × 0.10 × 0.08 mm

#### Data collection

Bruker–Nonius Roper CCD camera on κ-goniostat diffractometer
Absorption correction: multi-scan (*SADABS*; Sheldrick, 2007 ▶) *T*_min_ = 0.875, *T*_max_ = 0.991
12144 measured reflections
2984 independent reflections
1987 reflections with *I* > 2σ(*I*)
*R*_int_ = 0.070

#### Refinement

*R*[*F* ^2^ > 2σ(*F* ^2^)] = 0.082
*wR*(*F* ^2^) = 0.184
*S* = 1.10
2984 reflections
200 parameters
3 restraints
H atoms treated by a mixture of independent and constrained refinement
Δρ_max_ = 0.30 e Å^−3^
Δρ_min_ = −0.28 e Å^−3^

### 

Data collection: *COLLECT* (Hooft, 1998 ▶); cell refinement: *DENZO* (Otwinowski & Minor, 1997 ▶) and *COLLECT*; data reduction: *DENZO* and *COLLECT*; program(s) used to solve structure: *SHELXS97* (Sheldrick, 2008 ▶); program(s) used to refine structure: *SHELXL97* (Sheldrick, 2008 ▶); molecular graphics: *ORTEP-3* (Farrugia, 1997 ▶) and *DIAMOND* (Brandenburg, 2006 ▶); software used to prepare material for publication: *publCIF* (Westrip, 2010 ▶).

## Supplementary Material

Crystal structure: contains datablock(s) global, I. DOI: 10.1107/S1600536812005399/bt5815sup1.cif

Structure factors: contains datablock(s) I. DOI: 10.1107/S1600536812005399/bt5815Isup2.hkl

Supplementary material file. DOI: 10.1107/S1600536812005399/bt5815Isup3.cml

Additional supplementary materials:  crystallographic information; 3D view; checkCIF report

## Figures and Tables

**Table 1:** Hydrogen-bond geometry (Å, °)

*D*—H⋯*A*	*D*—H	H⋯*A*	*D*⋯*A*	*D*—H⋯*A*
O1—H1O⋯N1	0.84 (2)	1.79 (3)	2.534 (4)	147 (4)
N2—H2N⋯O3^i^	0.88 (2)	2.18 (3)	3.039 (4)	167 (2)
O2—H2O⋯O1^ii^	0.85 (4)	1.99 (4)	2.834 (3)	173 (4)
C14—H14⋯O4^i^	0.95	2.50	3.326 (4)	146

Symmetry codes: (i) 
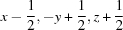
; (ii) 
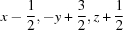
.

**Fig. 1.**
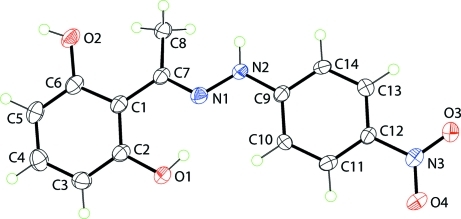
The molecular structure of (I) showing the atom-labelling scheme and displacement ellipsoids at the 50% probability level.

**Fig. 2.**
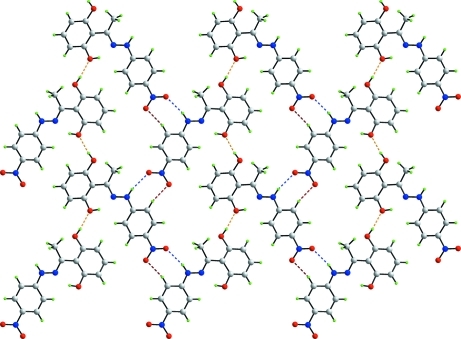
A plan view of the supramolecular layer in (I) sustained by O—H···O (orange dashed lines), N—H···O (blue dashed lines) and C—H···O (brown dashed lines) interactions.

**Fig. 3.**
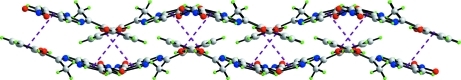
A side-on view of two supramolecular layers in (I) connected by π–π interactions (purple dashed lines).

**Fig. 4.**
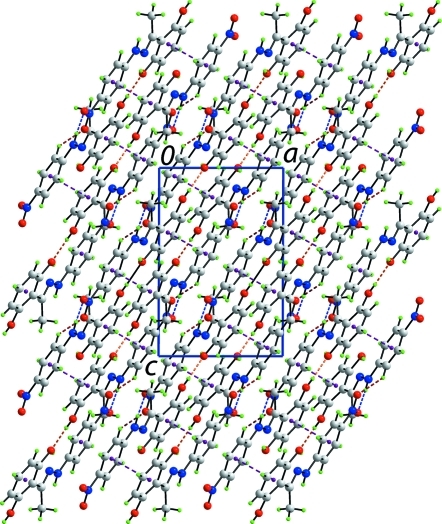
A view in projection down the b axis of the packing of supramolecular layers in (I).

## supplementary crystallographic information

### Comment

As a continuation of studies designed to ascertain the influence of substituents upon the supramolecular structures of hydrazones, in particular of those having potential biological activities, the title compound (*E*)-2,6-dihydroxyacetophenone 4-nitrophenylhydrazone (I) was investigated. Previous systematic investigations have included the study of substituted phenylhydrazines with substituted benzaldehydes (Glidewell *et al.*, 2004; Ferguson *et al.*, 2005) and 2-hydroxyacetophenone (Baddeley, de Souza França *et al.*, 2009). Hydrazones derived from substituted benzaldehydes and (pyrazinecarbonyl)hydrazine (Baddeley, Howie *et al.*, 2009; Howie, da Silva Lima *et al.*, 2010), 2-hydrazinyl-benzothiazole (Nogueira *et al.*, 2011), 7-chloroquinoline-4-hydrazide (Howie, de Souza *et al.*, 2010; de Souza *et al.*, 2010) and 2-hydrazinylacyl-N-isonicotine (Wardell *et al.*, 2007) have also been investigated along with *L*-serinyl derivatives, (*S*)-2-hydroxy-1-[*N*-(benzylidene)-hydrazinylcarbonyl]ethylcarbamate esters (Howie *et al.*, 2011).

In (I), Fig. 1, the dihedral angle between the benzene rings is 5.80 (16)°, indicating a planar molecule. The nitro group is co-planar with the benzene ring to which it is bonded as seen in the value of the O3—N3—C12—C11 torsion angle of -177.3 (3)°. The hydroxy group forms an intramolecular hydrogen bond with the imine-N1 atom, Table 1. The configuration about the N1═C7 imine bond [1.304 (4) Å] is *E*.

Supramolecular layers with an undulating topology in the (101) plane are formed by O—-H···O and N—H···O hydrogen bonds which are reinforced by C—H···O interactions, Fig. 2 and Table 1. Centrosymmetrically related layers are connected *via*π–π interactions occurring between the (C1–C6) and (C9–C14)^i^ rings [ring centroid···centroid distance = 3.5739 (19) Å, angle between rings = 5.80 (16)° for *i*: -*x*, 1 - *y*, 1 - *z*], Fig. 3. Layers stack without specific interactions between them, Fig. 4.

### Experimental

A solution of 4-nitrophenylhydrazine and 2,6-dihydroxyacetophenone (1 mmol each) in ethanol (25 ml) was refluxed for 1 h, rotary evaporated and the residue recrystallized from methanol, *M*.pt.: 501–503 K. IR (KBr, cm^-1^): ν 3600–2000 (v br), 3527, 3340, 1625, 1531. Anal. Found: C, 58.81; H, 4.86; N, 14.47. Calculated for C_14_H_13_N_3_O_4_: C, 58.53; H, 4.56; N, 14.62%.

### Refinement

The C-bound H atoms were geometrically placed (C—H = 0.95–0.98 Å) and refined as riding with *U*_iso_(H) = 1.2–1.5*U*_eq_(C). The O- and N-bound H atoms were located from a difference map and refined with the distance restraints O—H = 0.84±0.01 and N—H = 0.88±0.01 Å, and with *U*_iso_(H) = *zU*_eq_(carrier atom); *z* = 1.5 for O and *z* = 1.2 for N.

### Crystal data

**Table:**

C_14_H_13_N_3_O_4_	*F*(000) = 600
*M**_r_* = 287.27	*D*_x_ = 1.464 Mg m^−^^3^
Monoclinic, *P*2_1_/*n*	Mo *K*α radiation, λ = 0.71073 Å
Hall symbol: -P 2yn	Cell parameters from 8454 reflections
*a* = 7.9714 (3) Å	θ = 2.9–27.5°
*b* = 13.5021 (7) Å	µ = 0.11 mm^−^^1^
*c* = 12.1081 (5) Å	*T* = 120 K
β = 90.186 (3)°	Block, orange
*V* = 1303.20 (10) Å^3^	0.10 × 0.10 × 0.08 mm
*Z* = 4	

### Data collection

**Table:**

Bruker–Nonius Roper CCD camera on κ-goniostat diffractometer	2984 independent reflections
Radiation source: Bruker–Nonius FR591 rotating anode	1987 reflections with *I* > 2σ(*I*)
Graphite monochromator	*R*_int_ = 0.070
Detector resolution: 9.091 pixels mm^-1^	θ_max_ = 27.5°, θ_min_ = 3.0°
φ and ω scans	*h* = −10→8
Absorption correction: multi-scan (*SADABS*; Sheldrick, 2007)	*k* = −17→17
*T*_min_ = 0.875, *T*_max_ = 0.991	*l* = −15→15
12144 measured reflections	

### Refinement

**Table:**

Refinement on *F*^2^	Primary atom site location: structure-invariant direct methods
Least-squares matrix: full	Secondary atom site location: difference Fourier map
*R*[*F*^2^ > 2σ(*F*^2^)] = 0.082	Hydrogen site location: inferred from neighbouring sites
*wR*(*F*^2^) = 0.184	H atoms treated by a mixture of independent and constrained refinement
*S* = 1.10	*w* = 1/[σ^2^(*F*_o_^2^) + (0.0335*P*)^2^ + 2.8837*P*] where *P* = (*F*_o_^2^ + 2*F*_c_^2^)/3
2984 reflections	(Δ/σ)_max_ < 0.001
200 parameters	Δρ_max_ = 0.30 e Å^−^^3^
3 restraints	Δρ_min_ = −0.28 e Å^−^^3^

### Special details

**Table:**

Geometry. All s.u.'s (except the s.u. in the dihedral angle between two l.s. planes) are estimated using the full covariance matrix. The cell s.u.'s are taken into account individually in the estimation of s.u.'s in distances, angles and torsion angles; correlations between s.u.'s in cell parameters are only used when they are defined by crystal symmetry. An approximate (isotropic) treatment of cell s.u.'s is used for estimating s.u.'s involving l.s. planes.
Refinement. Refinement of *F*^2^ against ALL reflections. The weighted *R*-factor *wR* and goodness of fit *S* are based on *F*^2^, conventional *R*-factors *R* are based on *F*, with *F* set to zero for negative *F*^2^. The threshold expression of *F*^2^ > 2σ(*F*^2^) is used only for calculating *R*-factors(gt) *etc*. and is not relevant to the choice of reflections for refinement. *R*-factors based on *F*^2^ are statistically about twice as large as those based on *F*, and *R*- factors based on ALL data will be even larger.

### Fractional atomic coordinates and isotropic or equivalent isotropic displacement parameters (Å^2^)

**Table:**

	*x*	*y*	*z*	*U*_iso_*/*U*_eq_	
O1	0.1354 (3)	0.71070 (18)	0.50069 (19)	0.0328 (6)	
H1O	0.150 (5)	0.6502 (11)	0.514 (3)	0.049*	
O2	−0.1888 (3)	0.68611 (19)	0.83331 (18)	0.0340 (6)	
H2O	−0.241 (5)	0.721 (3)	0.880 (3)	0.051*	
O3	0.6044 (3)	0.19192 (18)	0.30034 (19)	0.0360 (6)	
O4	0.6016 (3)	0.32888 (19)	0.20547 (18)	0.0360 (6)	
N1	0.1233 (3)	0.5509 (2)	0.6101 (2)	0.0257 (6)	
N2	0.1909 (4)	0.4583 (2)	0.6225 (2)	0.0276 (6)	
H2N	0.167 (4)	0.423 (2)	0.6812 (19)	0.033*	
N3	0.5648 (3)	0.2804 (2)	0.2882 (2)	0.0273 (6)	
C1	−0.0115 (4)	0.6950 (2)	0.6750 (2)	0.0252 (7)	
C2	0.0357 (4)	0.7506 (3)	0.5807 (3)	0.0273 (7)	
C3	−0.0168 (5)	0.8477 (3)	0.5650 (3)	0.0334 (8)	
H3	0.0214	0.8845	0.5031	0.040*	
C4	−0.1245 (5)	0.8902 (3)	0.6400 (3)	0.0357 (8)	
H4	−0.1608	0.9566	0.6295	0.043*	
C5	−0.1806 (4)	0.8372 (3)	0.7305 (3)	0.0337 (8)	
H5	−0.2563	0.8670	0.7810	0.040*	
C6	−0.1266 (4)	0.7405 (3)	0.7478 (3)	0.0279 (7)	
C7	0.0573 (4)	0.5945 (2)	0.6957 (2)	0.0251 (7)	
C8	0.0634 (5)	0.5472 (3)	0.8079 (3)	0.0326 (8)	
H8A	−0.0272	0.4984	0.8141	0.049*	
H8B	0.0495	0.5981	0.8648	0.049*	
H8C	0.1718	0.5141	0.8181	0.049*	
C9	0.2810 (4)	0.4167 (2)	0.5372 (2)	0.0235 (7)	
C10	0.3233 (4)	0.4699 (2)	0.4419 (2)	0.0239 (7)	
H10	0.2865	0.5363	0.4329	0.029*	
C11	0.4188 (4)	0.4251 (2)	0.3614 (2)	0.0248 (7)	
H11	0.4489	0.4608	0.2968	0.030*	
C12	0.4704 (4)	0.3281 (2)	0.3751 (2)	0.0227 (7)	
C13	0.4297 (4)	0.2742 (3)	0.4688 (3)	0.0273 (7)	
H13	0.4659	0.2076	0.4768	0.033*	
C14	0.3360 (4)	0.3191 (2)	0.5497 (2)	0.0260 (7)	
H14	0.3084	0.2834	0.6148	0.031*	

### Atomic displacement parameters (Å^2^)

**Table:**

	*U*^11^	*U*^22^	*U*^33^	*U*^12^	*U*^13^	*U*^23^
O1	0.0374 (14)	0.0285 (13)	0.0327 (13)	0.0032 (11)	0.0141 (10)	0.0013 (11)
O2	0.0387 (14)	0.0374 (15)	0.0258 (12)	0.0003 (12)	0.0129 (10)	−0.0060 (11)
O3	0.0443 (15)	0.0266 (13)	0.0371 (13)	0.0044 (12)	0.0125 (11)	−0.0032 (11)
O4	0.0424 (15)	0.0411 (15)	0.0246 (12)	0.0021 (12)	0.0121 (10)	0.0022 (11)
N1	0.0265 (15)	0.0245 (15)	0.0261 (13)	−0.0025 (12)	0.0036 (11)	−0.0012 (11)
N2	0.0351 (16)	0.0240 (15)	0.0238 (13)	0.0011 (12)	0.0106 (11)	0.0000 (11)
N3	0.0250 (14)	0.0327 (16)	0.0243 (13)	0.0006 (12)	0.0029 (11)	−0.0028 (12)
C1	0.0226 (16)	0.0266 (17)	0.0263 (15)	−0.0051 (13)	0.0035 (12)	−0.0053 (13)
C2	0.0293 (18)	0.0277 (18)	0.0251 (15)	−0.0045 (15)	0.0051 (13)	−0.0038 (13)
C3	0.043 (2)	0.0268 (19)	0.0308 (18)	−0.0026 (16)	0.0061 (15)	0.0012 (14)
C4	0.042 (2)	0.0274 (19)	0.0380 (19)	0.0026 (16)	−0.0010 (16)	−0.0075 (15)
C5	0.034 (2)	0.038 (2)	0.0289 (17)	0.0035 (16)	0.0042 (14)	−0.0112 (15)
C6	0.0280 (18)	0.0318 (19)	0.0239 (15)	−0.0044 (15)	0.0020 (13)	−0.0081 (14)
C7	0.0245 (17)	0.0243 (17)	0.0264 (16)	−0.0064 (13)	0.0061 (13)	−0.0061 (13)
C8	0.045 (2)	0.0270 (18)	0.0264 (17)	−0.0003 (16)	0.0109 (15)	−0.0036 (14)
C9	0.0235 (16)	0.0247 (17)	0.0224 (14)	−0.0024 (13)	0.0041 (12)	−0.0033 (13)
C10	0.0305 (18)	0.0170 (16)	0.0244 (15)	0.0013 (13)	0.0037 (13)	0.0008 (12)
C11	0.0268 (17)	0.0288 (18)	0.0188 (14)	−0.0039 (14)	0.0034 (12)	0.0004 (13)
C12	0.0221 (16)	0.0246 (16)	0.0215 (14)	−0.0011 (13)	0.0033 (12)	−0.0042 (12)
C13	0.0300 (18)	0.0239 (17)	0.0281 (16)	0.0030 (14)	0.0021 (13)	0.0009 (13)
C14	0.0308 (18)	0.0259 (17)	0.0213 (15)	−0.0026 (14)	0.0078 (13)	0.0033 (13)

### Geometric parameters (Å, º)

**Table:**

O1—C2	1.366 (4)	C4—H4	0.9500
O1—H1O	0.841 (10)	C5—C6	1.390 (5)
O2—C6	1.364 (4)	C5—H5	0.9500
O2—H2O	0.844 (10)	C7—C8	1.503 (4)
O3—N3	1.244 (4)	C8—H8A	0.9800
O4—N3	1.233 (3)	C8—H8B	0.9800
N1—C7	1.304 (4)	C8—H8C	0.9800
N1—N2	1.369 (4)	C9—C14	1.398 (4)
N2—C9	1.380 (4)	C9—C10	1.401 (4)
N2—H2N	0.880 (10)	C10—C11	1.378 (4)
N3—C12	1.447 (4)	C10—H10	0.9500
C1—C6	1.414 (4)	C11—C12	1.383 (4)
C1—C2	1.418 (4)	C11—H11	0.9500
C1—C7	1.484 (5)	C12—C13	1.387 (4)
C2—C3	1.390 (5)	C13—C14	1.375 (4)
C3—C4	1.377 (5)	C13—H13	0.9500
C3—H3	0.9500	C14—H14	0.9500
C4—C5	1.385 (5)		
			
C2—O1—H1O	109 (3)	N1—C7—C1	115.4 (3)
C6—O2—H2O	113 (3)	N1—C7—C8	121.0 (3)
C7—N1—N2	119.0 (3)	C1—C7—C8	123.5 (3)
N1—N2—C9	119.7 (3)	C7—C8—H8A	109.5
N1—N2—H2N	120 (2)	C7—C8—H8B	109.5
C9—N2—H2N	120 (2)	H8A—C8—H8B	109.5
O4—N3—O3	123.0 (3)	C7—C8—H8C	109.5
O4—N3—C12	118.7 (3)	H8A—C8—H8C	109.5
O3—N3—C12	118.3 (3)	H8B—C8—H8C	109.5
C6—C1—C2	116.5 (3)	N2—C9—C14	117.8 (3)
C6—C1—C7	122.2 (3)	N2—C9—C10	122.4 (3)
C2—C1—C7	121.4 (3)	C14—C9—C10	119.7 (3)
O1—C2—C3	116.8 (3)	C11—C10—C9	119.6 (3)
O1—C2—C1	121.3 (3)	C11—C10—H10	120.2
C3—C2—C1	122.0 (3)	C9—C10—H10	120.2
C4—C3—C2	119.4 (3)	C10—C11—C12	119.7 (3)
C4—C3—H3	120.3	C10—C11—H11	120.2
C2—C3—H3	120.3	C12—C11—H11	120.2
C3—C4—C5	120.7 (4)	C11—C12—C13	121.6 (3)
C3—C4—H4	119.7	C11—C12—N3	119.4 (3)
C5—C4—H4	119.7	C13—C12—N3	119.0 (3)
C6—C5—C4	120.3 (3)	C14—C13—C12	118.8 (3)
C6—C5—H5	119.9	C14—C13—H13	120.6
C4—C5—H5	119.9	C12—C13—H13	120.6
O2—C6—C5	120.4 (3)	C13—C14—C9	120.6 (3)
O2—C6—C1	118.5 (3)	C13—C14—H14	119.7
C5—C6—C1	121.0 (3)	C9—C14—H14	119.7
			
C7—N1—N2—C9	−171.3 (3)	C6—C1—C7—C8	−22.2 (5)
C6—C1—C2—O1	−174.7 (3)	C2—C1—C7—C8	157.6 (3)
C7—C1—C2—O1	5.5 (5)	N1—N2—C9—C14	−174.8 (3)
C6—C1—C2—C3	5.3 (5)	N1—N2—C9—C10	7.6 (5)
C7—C1—C2—C3	−174.5 (3)	N2—C9—C10—C11	177.7 (3)
O1—C2—C3—C4	176.7 (3)	C14—C9—C10—C11	0.1 (5)
C1—C2—C3—C4	−3.3 (5)	C9—C10—C11—C12	0.6 (5)
C2—C3—C4—C5	0.1 (5)	C10—C11—C12—C13	−0.5 (5)
C3—C4—C5—C6	1.0 (5)	C10—C11—C12—N3	177.5 (3)
C4—C5—C6—O2	−176.3 (3)	O4—N3—C12—C11	2.4 (4)
C4—C5—C6—C1	1.2 (5)	O3—N3—C12—C11	−177.3 (3)
C2—C1—C6—O2	173.3 (3)	O4—N3—C12—C13	−179.5 (3)
C7—C1—C6—O2	−6.8 (5)	O3—N3—C12—C13	0.9 (4)
C2—C1—C6—C5	−4.2 (5)	C11—C12—C13—C14	−0.2 (5)
C7—C1—C6—C5	175.6 (3)	N3—C12—C13—C14	−178.2 (3)
N2—N1—C7—C1	179.4 (3)	C12—C13—C14—C9	0.8 (5)
N2—N1—C7—C8	3.4 (5)	N2—C9—C14—C13	−178.5 (3)
C6—C1—C7—N1	161.9 (3)	C10—C9—C14—C13	−0.8 (5)
C2—C1—C7—N1	−18.3 (4)		

### Hydrogen-bond geometry (Å, º)

**Table:**

*D*—H···*A*	*D*—H	H···*A*	*D*···*A*	*D*—H···*A*
O1—H1*O*···N1	0.84 (2)	1.79 (3)	2.534 (4)	147 (4)
N2—H2*N*···O3^i^	0.88 (2)	2.18 (3)	3.039 (4)	167 (2)
O2—H2*O*···O1^ii^	0.85 (4)	1.99 (4)	2.834 (3)	173 (4)
C14—H14···O4^i^	0.95	2.50	3.326 (4)	146

Symmetry codes: (i) *x*−1/2, −*y*+1/2, *z*+1/2; (ii) *x*−1/2, −*y*+3/2, *z*+1/2.
